# Risk factors for multidrug-resistant tuberculosis: a predictive model study

**DOI:** 10.3389/fmed.2024.1410690

**Published:** 2024-09-27

**Authors:** Lianpeng Wu, Xiaoxiao Cai, Xiangao Jiang

**Affiliations:** ^1^Department of Clinical Laboratory Medicine, Wenzhou Central Hospital, The Ding Li Clinical College of Wenzhou Medical University, Wenzhou, China; ^2^Key Laboratory of Diagnosis and Treatment of New and Recurrent Infectious Diseases of Wenzhou, Wenzhou Central Hospital, The Dingli Clinical College of Wenzhou Medical University, Wenzhou, China; ^3^Department of Clinical Laboratory Medicine, Wenzhou People's Hospital, Wenzhou, China; ^4^Department of Infectious Diseases, Wenzhou Central Hospital, The Ding Li Clinical College of Wenzhou Medical University, Wenzhou, China

**Keywords:** drug-resistant tuberculosis, multidrug-resistant tuberculosis, risk factors, nomogram model, predictive value

## Abstract

**Objective:**

To investigate the risk factors associated with Multidrug-resistant tuberculosis (MDR-TB) in people with drug-resistant tuberculosis (DR-TB) and develop a predictive model.

**Methods:**

A total of 893 individuals with DR-TB treated at Wenzhou Central Hospital from January 2018 to December 2022 were included in the study after excluding 178 individuals with incomplete clinical and laboratory data, leaving 715 individuals for analysis. Data on demographic information, baseline clinical characteristics, laboratory and imaging results, and clinical diagnosis were collected to identify the risk factors for MDR-TB and establish a predictive model.

**Results:**

Multivariate logistic regression analysis identified residence in rural areas, retreatment of TB, presence of pulmonary cavity, uric acid (UA) ≥ 346 μmol/L and c-reactive protein (CRP) < 37.3 mg/L as independent risk factors for MDR-TB in individuals with DR-TB. A nomogram model was constructed using these five factors to predict the risk of MDR-TB, with an area under the ROC curve (AUC) of 0.758 for the training group and 0.775 for the validation group. Calibration curve analysis showed good agreement between predicted and actual MDR-TB incidence in both groups, and decision curve analysis showed that the nomogram model had a higher rate of clinical net benefit.

**Conclusion:**

This study suggests that residence, types of TB treatment, presence of pulmonary cavity, UA and CRP are associated with MDR-TB occurrence in individuals with DR-TB, and the nomogram model developed in this study shows promising predictive value.

## 1 Introduction

Tuberculosis (TB) is a chronic infectious disease caused by *Mycobacterium tuberculosis* (MTB) infection (1). The 2023 global TB report from the World Health Organization (WHO) revealed that approximately 1.3 million individuals worldwide succumbed to TB in 2022 (2). TB remains the second leading cause of death from a single infectious agent globally, presenting significant challenges to public health (3). The emergence of drug-resistant tuberculosis (DR-TB), particularly multidrug-resistant tuberculosis (MDR-TB), is a significant contributor to mortality (4). A study on the global prevalence of MDR-TB reported a standardized incidence rate of 8.26/100,000 and a total of 100,000 MDR-TB cases worldwide (5). In comparison to regular TB, MDR-TB is more challenging to treat, requires longer treatment periods, comes with higher costs, leads to more adverse reactions, and has a lower cure rate (6, 7). Early identification of risk factors for MDR-TB can decrease the likelihood of developing MDR-TB and aid in prompt diagnosis and treatment (8). Admassu et al. (9) conducted a study in southern Ethiopia which revealed that direct contact with individuals with TB, previous TB treatment history, smoking, and rural residence were identified as risk factors for MDR-TB. Similarly, a study in Serbia by Stosic et al. (10) found that factors such as monthly household income, lack of treatment, TB-related stigma, subjective grief, use of sedatives, and chronic obstructive pulmonary disease were associated with MDR-TB occurrence. Another study by Sambas et al. (11) in Mecca, Saudi Arabia highlighted age, smoking, lung disease, and previous TB history as important factors related to MDR-TB. Huai et al. (12) conducted a study on individuals with TB in China and discovered that being registered as a floating population, having more than 3 pulmonary lesions, undergoing the first treatment for over 8 months, having a history of more than 3 anti-TB treatments, experiencing treatment failure or continuous deterioration, and residing in the same area for less than 30 years were linked to MDR-TB occurrence. This study focused on individuals with DR-TB admitted to Wenzhou Central Hospital between January 2018 and December 2022. It analyzed the risk factors for MDR-TB in individuals with DR-TB and developed a prediction model to assist in clinical diagnosis, treatment, and precise prevention and control strategies.

## 2 Materials and methods

### 2.1 Study subject selection

**Figure 1:**
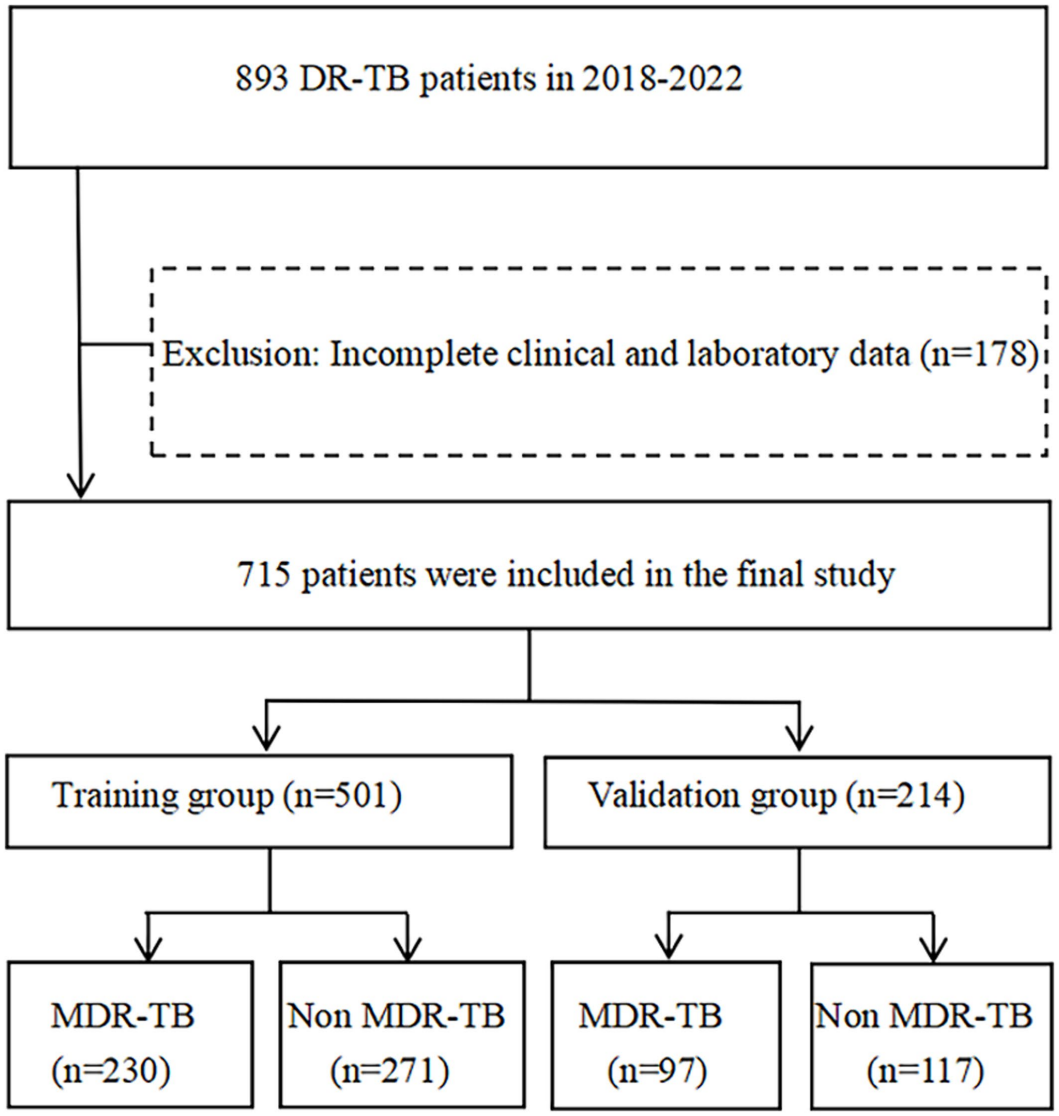
Flow chart of the subjects enrolled in the study. DR-TB, drug-resistant tuberculosis; MDR-TB, multidrug-resistant tuberculosis.

A total of 893 individuals with DR-TB were admitted to Wenzhou Central Hospital between January 2018 and December 2022. The diagnostic criteria for DR-TB were based on phenotypic drug sensitivity tests conducted on culture-positive strains. After excluding 178 individuals due to incomplete data, including missing clinical and laboratory information, the final sample size for this study comprised 715 individuals. These individuals were then randomly assigned to either a training group (*n* = 501) and a verification group (*n* = 214) at a ratio of 7:3. The training group comprised 230 individuals with MDR-TB and 271 individuals with non-MDR-TB, while the verification group included 97 MDR-TB individuals and 117 non-MDR-TB individuals (Figure 1). The sample size was calculated using MedCalc software. The ratio of participants in the study group to those in the control group was set at 1:1, with a power of 0.9. Under a bilateral alpha level of 0.05, a minimum of 143 cases was required in each group to achieve the expected effect (AUC = 0.75). The sample size for this study met the minimum requirement. This study has been approved by the hospital medical ethics (batch No. L2024-01-059), utilising retrospective and anonymous data collection methods, which did not involve patient privacy. All experiments were carried out in compliance with relevant laws and guidelines and with the ethical standards of the Declaration of Helsinki.

### 2.2 Data collection

The medical records of 715 individuals with DR-TB were reviewed. The clinical information was collected retrospectively, including the demographic information (age, sex, marriage, nationality, occupation, migration status, and residence), baseline clinical characteristics (fever, cough, expectoration, hemoptysis, thoracodynia, chest tightness, fatigue, emaciation, night sweats), laboratory tests (Blood routine examination, biochemical examination, HIV antibody test, acid-fast smear and drug sensitivity test of MTB), computed tomography imaging examination, and clinical diagnosis.

### 2.3 Definitions and classifications of TB treatments and drug resistance

DR-TB is defined as resistance to at least one anti-TB drug. MDR-TB is defined as resistance to at least both isoniazid and rifampicin.

Initial treatment TB refers to three subcategories: (a) individuals who have never received anti-TB drugs for TB, (b) individuals who have not completed the entire course of standard chemotherapy treatment, and (c) individuals who have received irregular chemotherapy for less than 1 month.

Retreatment TB refers to two subcategories: (a) individuals who have been treated with anti-TB drugs for more than 1 month due to unreasonable or irregular use, and (b) individuals who have experienced initial treatment failure and recurrence.

### 2.4 Statistical analysis

Statistical analyses were performed using SPSS software (version 26.0, IBM, New York, USA). The counting data were presented as frequency and percentage. The patient’s laboratory test results were dichotomized using the mean as the cut-off value, and comparisons of classified variables was done using the chi-square test. The data were randomly divided into a training group and a verification group at a ratio of 7:3. Univariate and multivariate logistic regression analyses were conducted to explore the risk factors related to MDR-TB, with Spearman correlation used to examine the relationships between these factors. A nomogram model for predicting MDR-TB was developed using R software and rms package (version 4.1.5, R Foundation for Statistical Computing, Vienna, Austria). The predictive performance of the model was assessed through receiver operating characteristic curve (ROC), calibration curve, and decision curve analysis (DCA). A significance level of *p-*value < 0.05 was considered statistically significant.

## 3 Results

### 3.1 Study subjects’ characteristics

In this study, a total of 715 subjects with DR-TB were randomly divided into a training group (*n* = 501) and a verification group (*n* = 214). There were no statistically significant differences in variables between the two groups (*p* > 0.05). Among the subject population, 74.68% were male and 25.32% were female, with the highest proportion falling in the age range of 45 to 59 (26.99%). The majority of individuals with DR-TB identified as Han nationality (97.20%), married (74.26%), unemployed (89.65%), and local residents (75.38%). In terms of TB types, 95.10% had pulmonary tuberculosis (PTB), 0.84% had extrapulmonary tuberculosis (EPTB), and 4.06% had both. Regarding treatment, 77.48% were on initial treatment while 22.52% were on retreatment. A portion of subjects had a history of smoking (29.37%) and drinking (18.32%). Clinical symptoms included fever (25.31%), cough (76.36%), expectoration (66.71%), hemoptysis (16.22%), thoracodynia (9.37%), chest tightness (14.55%), fatigue (12.59%), emaciation (17.34%), night sweats (8.67%), and pulmonary cavity (53.15%). Acid-fast smear (AFS) was positive in 52.17% of subjects, and nearly half had MDR-TB (45.73%). Additional baseline data can be found in Table 1.

**Table 1:** The demographic and clinical parameters of individuals with DR-TB.

Variables		Total [*n* = 715 (%)]	Training group [*n* = 501 (%)]	Validation group [*n* = 214 (%)]	*p*-value
Gender	Male	534 (74.68)	365 (72.85)	169 (78.97)	0.085
	Female	181 (25.32)	136 (27.15)	45 (21.03)	
Age	12 ~ 30	166 (23.22)	108 (21.56)	58 (27.10)	0.090
	31 ~ 44	164 (22.94)	127 (25.35)	37 (17.29)	
	45 ~ 59	193 (26.99)	132 (26.35)	61 (28.51)	
	60~	192 (26.85)	134 (26.74)	58 (27.10)	
Marital status	Unmarried	148 (20.70)	97 (19.36)	51 (23.83)	0.232
	Married	531 (74.26)	376 (75.05)	155 (72.43)	
	Divorce	19 (2.66)	13 (2.60)	6 (2.80)	
	Widowed	17 (2.38)	15 (2.99)	2 (0.94)	
Ethnicity	Han	695 (97.20)	484 (96.61)	211 (98.60)	0.139
	Others	20 (2.80)	17 (3.39)	3 (1.40)	
Occupation	Unemployed	641 (89.65)	442 (88.22)	199 (92.99)	0.055
	Employed	74 (10.35)	59 (11.78)	15 (7.01)	
Migrant	Yes	176 (24.62)	123 (24.55)	53 (24.77)	0.951
	No	539 (75.38)	378 (75.45)	161 (75.23)	
Residence	Urban	367 (51.33)	261 (52.10)	106 (49.53)	0.530
	Rural	348 (48.67)	240 (47.90)	108 (50.47)	
Types of TB treatment	Initial treatment	554 (77.48)	389 (77.65)	165 (77.10)	0.874
	Retreatment	161 (22.52)	112 (22.35)	49 (22.90)	
Types of TB	PTB	680 (95.10)	478 (95.41)	202 (94.39)	0.495
	EPTB	6 (0.84)	5 (1.00)	1 (0.47)	
	PTB combined with EPTB	29 (4.06)	18 (3.59)	11 (5.14)	
ESR, mm/h	≥39	323 (45.18)	226 (45.11)	97 (45.33)	0.957
WBC count, 10^9^/L	≥6.9	323 (45.18)	222 (44.31)	101 (47.20)	0.478
NEUT count, 10^9^/L	≥4.9	306 (42.80)	209 (41.72)	97 (45.33)	0.372
LYM count, 10^9^/L	≥1.3	363 (50.77)	257 (51.30)	106 (49.53)	0.666
HGB, g/L	≥124	374 (52.31)	256 (51.10)	118 (55.14)	0.322
PLT, 10^9^/L	≥276	326 (45.59)	229 (45.71)	97 (45.33)	0.925
ALT, U/L	≥22	194 (27.13)	138 (27.55)	56 (26.17)	0.705
ALB, g/L	≥36.4	366 (51.19)	253 (50.50)	113 (52.80)	0.572
FPG, mmol/L	≥6.1	188 (26.29)	137 (27.35)	51 (23.83)	0.328
Cr, μmol/L	≥70	289 (40.42)	203 (40.52)	86 (40.19)	0.934
UA, μmol/L	≥346	289 (40.42)	193 (38.52)	96 (44.86)	0.114
TC, mmol/L	≥3.82	306 (42.80)	215 (42.91)	91 (42.52)	0.923
TG, mmol/L	≥1.10	267 (37.34)	187 (37.33)	80 (37.38)	0.988
CRP, mg/L	≥37.3	266 (37.20)	189 (37.73)	77 (35.98)	0.659
HIV infection	Negative	701 (98.00)	491 (54.29)	210 (98.13)	0.911
	Positive	14 (2.00)	10 (45.71)	4 (1.87)	
AFS	Negative	373 (52.17)	272 (54.29)	101 (47.20)	0.082
	Positive	342 (47.83)	229 (45.71)	113 (52.80)	
MDR-TB	Yes	327 (45.73)	97 (45.33)	230 (45.91)	0.886
	No	388 (54.27)	117 (54.67)	271 (54.09)	
Smoking history		210 (29.37)	139 (27.75)	71 (33.18)	0.144
History of drinking		131 (18.32)	83 (16.57)	48 (22.43)	0.063
Fever		181 (25.31)	128 (25.55)	53 (24.77)	0.826
Cough		546 (76.36)	379 (75.649)	167 (78.037)	0.491
Expectoration		477 (66.71)	335 (66.87)	142 (66.36)	0.894
Hemoptysis		116 (16.22)	76 (15.17)	40 (18.69)	0.242
Thoracodynia		67 (9.37)	48 (9.58)	19 (8.88)	0.768
Chest tightness		104 (14.55)	73 (14.57)	31 (14.49)	0.976
Fatigue		90 (12.59)	61 (12.18)	29 (13.55)	0.612
Emaciation		124 (17.34)	91 (18.16)	33 (15.42)	0.375
Night sweats		62 (8.67)	41 (8.18)	21 (9.81)	0.478
Pulmonary cavity		380 (53.15)	266 (53.09)	114 (53.27)	0.965

TB, tuberculosis; PTB, pulmonary tuberculosis; EPTB, extrapulmonary tuberculosis; ESR, erythrocyte sedimentation rate; WBC, white blood cell; NEUT, neutrophil; LYM, lymphocyte; HGB, haemoglobin; PLT, platelet; ALT, alanine aminotransferase; ALB, albumin; FPG, fasting plasma glucose; Cr, creatinine; UA, uric acid; TC, total cholesterol; TG, triglyceride; CRP, C-reactive protein; HIV, human immunodeficiency virus; AFS, acid fast smear; MDR-TB, multidrug-resistant tuberculosis.

### 3.2 Univariate and multivariate analysis of MDR-TB in individuals with DR-TB

Univariate analysis of demographic information and clinical parameters identified residence, types of TB treatment, fever, cough, pulmonary cavity, lymphocyte count, UA, and CRP as influencing factors for MDR-TB in individuals with DR-TB (Table 2). Subsequently, multivariate logistic regression analysis was conducted to assess the risk factors for MDR-TB while adjusting for confounding variables. The results revealed that the risk of MDR-TB in rural residents is 2.135 times higher than in urban residents (OR = 2.135, 95% CI: 1.440–3.180, *p* < 0.01). Additionally, the risk of MDR-TB in retreated individuals with TB is 3.940 times higher than in initially treated individuals (OR = 3.940, 95% CI: 2.387–6.650, *p* < 0.01). The risk of MDR-TB in individuals with pulmonary cavity is 2.736 times higher than in individuals without pulmonary cavity (OR = 2.736, 95% CI: 1.832–4.115, *p* < 0.01). Moreover, the risk of MDR-TB in individuals with UA ≥ 346 μmol/L is 1.681 times higher than in individuals with UA < 346 μmol/L (OR = 1.681, 95% CI: 1.104–2.569, *p* < 0.05). Lastly, the risk of developing MDR-TB in individuals with CRP < 37.3 mg/L is 1.668 times higher than in individuals with CRP ≥ 37.3 mg/L (OR = 1.668, 95% CI: 1.068–2.619, *p* < 0.05; Table 2).

**Table 2:** Univariate and multivariate logistic regression analysis of DR-TB individuals with MDR-TB.

Variables		Univariate analysis		Multivariate analysis	
		OR (95% CI)	*p*-value	OR (95% CI)	*p*-value
Gender	Male	Reference			
	Female	0.869 (0.585–1.292)	0.489		
Age	12 ~ 30	Reference			
	31 ~ 44	1.655 (0.987–2.777)	0.056		
	45 ~ 59	0.933 (0.557–1.563)	0.792		
	60~	1.093 (0.655–1.822)	0.734		
Marital status	Married	Reference			
	Unmarried	0.943 (0.603–1.477)	0.799		
	Divorce	0.758 (0.264–2.171)	0.605		
	Widowed	0.505 (0.153–1.669)	0.263		
Ethnicity	Han	Reference			
	Others	0.480 (0.166–1.382)	0.174		
Occupation	Employed	Reference			
	Unemployed	0.725 (0.416–1.264)	0.257		
Migrant	No	Reference			
	Yes	0.898 (0.596–1.353)	0.607		
Residence	Urban	Reference		Reference	
	Rural	2.323 (1.622–3.327)	<0.001	2.135 (1.440–3.180)	<0.001
Types of TB treatment	Initial treatment	Reference		Reference	
	Retreatment	4.993 (3.107–8.024)	<0.001	3.940 (2.387–6.650)	<0.001
Types of TB	PTB	Reference			
	EPTB	0.283 (0.031–2.555)	0.261		
	PTB combined with EPTB	0.436 (0.153–1.242)	0.120		
Smoking history	No	Reference			
	Yes	1.049 (0.709–1.552)	0.812		
History of drinking	No	Reference			
	Yes	0.697 (0.431–1.129)	0.142		
Fever	No	Reference			
	Yes	0.783 (0.521–1.175)	0.237		
Cough	No	Reference		Reference	
	Yes	1.708 (1.122–2.602)	0.013	1.571 (0.705–3.493)	0.267
Expectoration	No	Reference		Reference	
	Yes	1.508 (1.033–2.201)	0.033	0.973 (0.471–2.021)	0.940
Hemoptysis	No	Reference			
	Yes	1.291 (0.792–2.105)	0.305		
Thoracodynia	No	Reference			
	Yes	1.315 (0.725–2.386)	0.368		
Chest tightness	No	Reference			
	Yes	1.620 (0.983–2.672)	0.059		
Fatigue	No	Reference			
	Yes	0.737 (0.426–1.273)	0.274		
Emaciation	No	Reference			
	Yes	0.729 (0.459–1.158)	0.180		
Night sweats	No	Reference			
	Yes	0.916 (0.481–1.742)	0.788		
Pulmonary cavity	No	Reference		Reference	
	Yes	3.196 (2.210–4.622)	<0.001	2.736 (1.832–4.115)	<0.001
ESR, mm/h	<39	Reference			
	≥39	0.704 (0.494–1.005)	0.053		
WBC count, 10^9^/L	<6.9	Reference			
	≥6.9	1.142 (0.802–1.627)	0.461		
NEUT count, 10^9^/L	<4.9	Reference			
	≥4.9	1.070 (0.749–1.528)	0.709		
LYM count, 10^9^/L	<1.3	Reference		Reference	
	≥1.3	1.427 (1.002–2.031)	0.048	1.074 (0.707–1.631)	0.736
HGB, 10^9^/L	<124	Reference			
	≥124	1.272 (0.895–1.809)	0.180		
PLT, 10^9^/L	<276	Reference			
	≥276	0.904 (0.635–1.286)	0.573		
ALT, U/L	<22	Reference			
	≥22	0.712 (0.478–1.060)	0.094		
ALB, g/L	<36.4	Reference			
	≥36.4	1.132 (0.796–1.609)	0.490		
FPG, mmol/L	<6.1	Reference			
	≥6.1	0.854 (0.575–1.268)	0.434		
Cr, μmol/L	<70	Reference			
	≥70	1.341 (0.937–1.919)	0.108		
UA, μmol/L	<346	Reference		Reference	
	≥346	1.808 (1.257–2.601)	0.001	1.681 (1.104–2.569)	0.016
TC, mmol/L	<3.82	Reference			
	≥3.82	1.229 (0.862–1.754)	0.254		
TG, mmol/L	<1.10	Reference			
	≥1.10	1.417 (0.985–2.039)	0.060		
CRP, mg/L	≥ 37.3	Reference		Reference	
	<37.3	1.554 (0.446–0.929)	0.018	1.668 (1.068–2.619)	0.025
HIV infection	Negative	Reference			
	Positive	0.498 (0.127–1.950)	0.317		
AFS	Negative	Reference			
	Positive	1.249 (0.878–1.778)	0.217		

TB, tuberculosis; PTB, pulmonary tuberculosis; EPTB, extrapulmonary tuberculosis; ESR, erythrocyte sedimentation rate; WBC, white blood cell; NEUT, neutrophil; LYM, lymphocyte; HGB, haemoglobin; PLT, platelet; ALT, alanine aminotransferase; ALB, albumin; FPG, fasting plasma glucose; Cr, creatinine; UA, uric acid; TC, total cholesterol; TG, triglyceride; CRP, C-reactive protein; HIV, human immunodeficiency virus; AFS, acid fast smear.

### 3.3 Prediction model construction and evaluation

**Figure 2:**
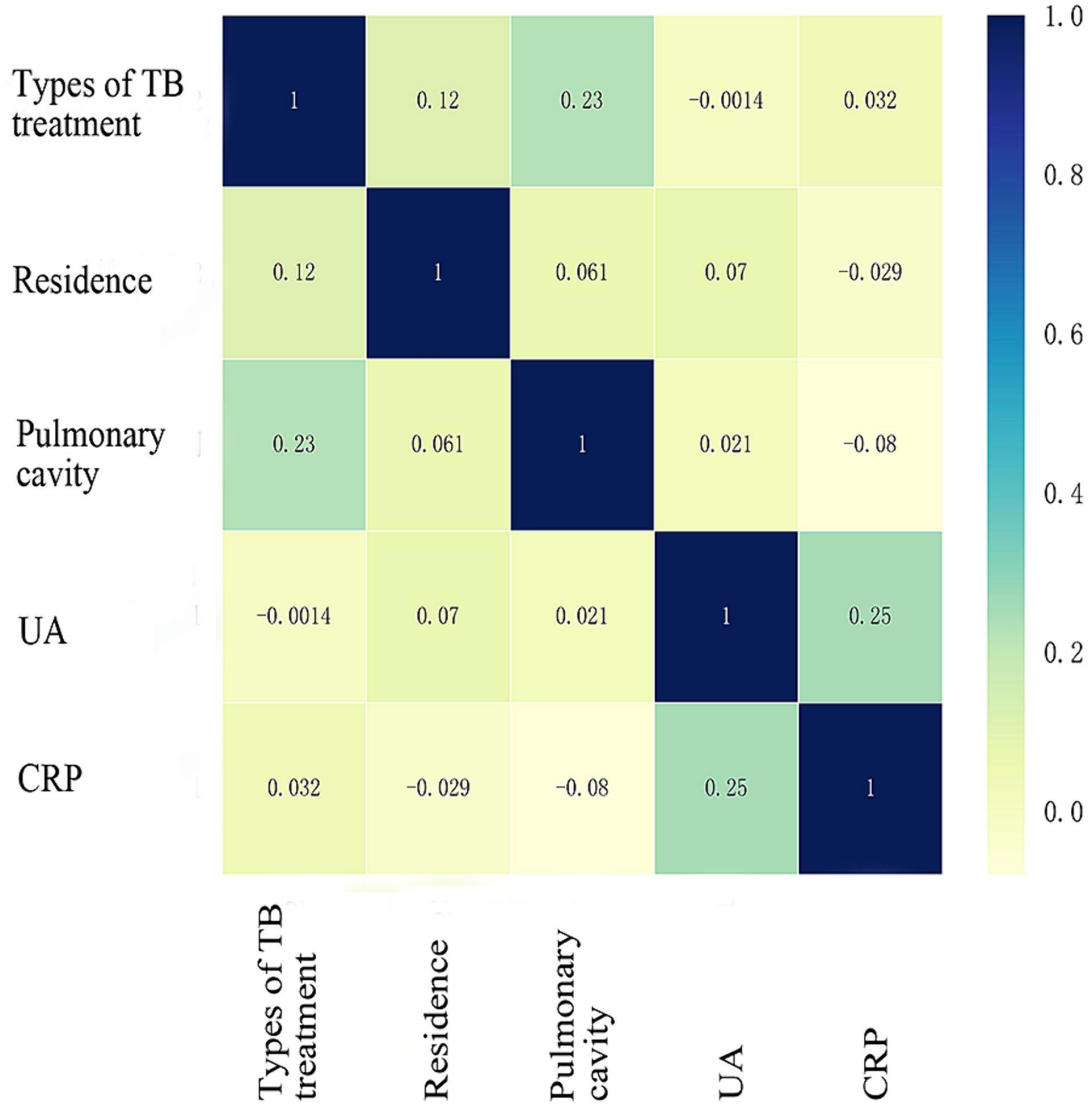
Spearman correlation coefficient of five risk factors. TB, Tuberculosis; UA, Uric acid; CRP, C-reactive protein. Units of UA (μmol/L) and CRP (mg/L), spelman correlation analysis was performed after UA was classified according to whether or not ≥346 μmol/L and CRP according to whether ≥37.3 mg/L.

**Figure 3:**
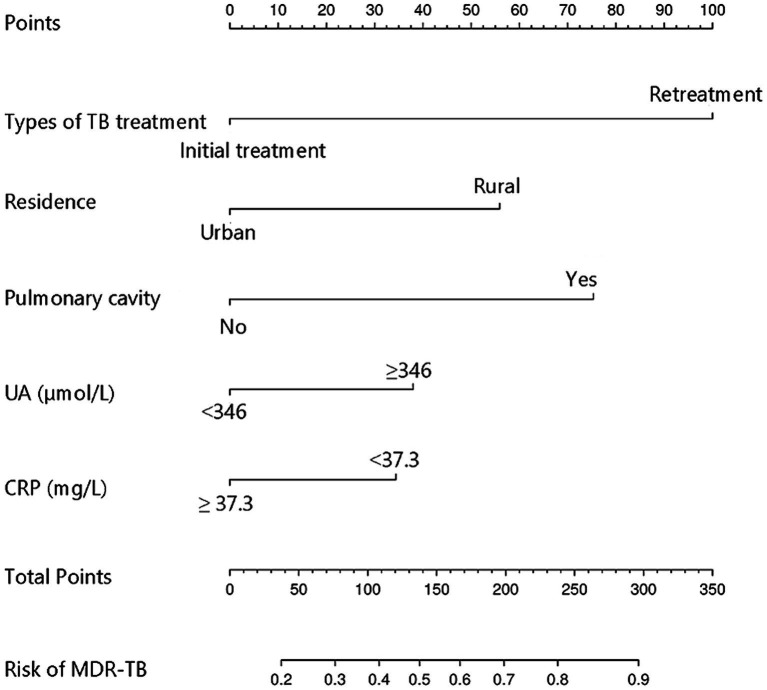
Nomogram model for predicting the development of MDR-TB in individuals with DR-TB. TB, Tuberculosis; UA, Uric acid; CRP, C-reactive protein; MDR-TB, Multidrug-resistant tuberculosis; DR-TB, Drug-resistant tuberculosis.

**Figure 4:**
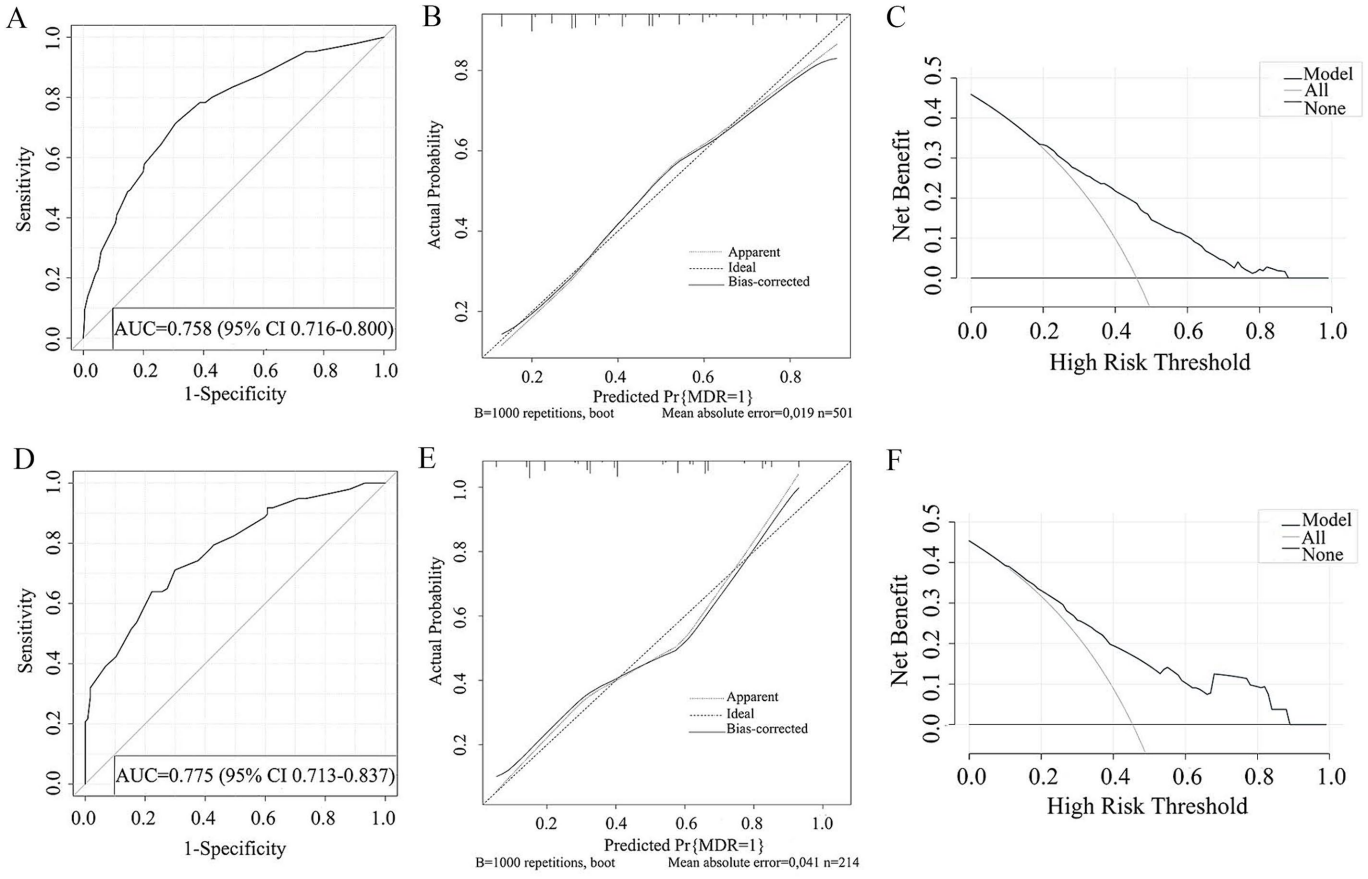
ROC curve **(A)**, Calibration curve **(B)**, DCA curve **(C)** of the prediction model developed in the training cohorts. ROC curve **(D)**, Calibration curve **(E)**, DCA curve **(F)** of the prediction model developed in the validation cohorts.

The study examined five selected risk factors (residence, types of TB treatment, pulmonary cavity, UA, CRP) through spearman correlation analysis. Results indicated no significant correlation among these factors (all R < 0.40; Figure 2). A nomogram model was developed for predicting MDR-TB occurrence based on these factors (Figure 3). The AUC of the training group was 0.758 (95%CI = 0.716 ~ 0.800), illustrated in Figure 4A, indicating the specific good differentiation of the model. As shown in Figure 4B, the calibration curve analysis using bootstrap method showed that the calibration curve was closed to the standard curve, and the Hosmer-Lemeshow test results showed that the goodness of fit of the prediction model was good (*p* = 0.979). DCA curve results indicated a higher clinical net benefit rate for the model, as shown in Figure 4C. Validation with a separate group yielded an AUC of 0.775 (95% CI = 0.713 ~ 0.837; Figure 4D), indicating the specific good differentiation of the model. The calibration curve for the validation group also showed that the calibration curve was close to the standard curve (Figure 4E), and the Hosmer-Lemeshow test results showed that the goodness of fit of the prediction model was good (*p* = 0.735). The DAC curve results also showed that the model had a higher clinical net benefit rate, as shown in Figure 4F.

## 4 Discussion

The target population of this study comprises individuals with DR-TB. Through univariate and multivariate Logistic regression analysis, combined with demographic information and clinical indicators, five variables were selected to predict the risk of MDR-TB, namely residence, type of TB treatment, pulmonary cavity, UA and CRP. Previous studies have identified gender, age, nationality, migrant status, residence, types of TB treatment, smoking history, pulmonary cavity, and AFS results as statistically significant factors associated with MDR-TB occurrence (9, 13–17). However, in this study, gender, age, nationality, migrant status, smoking history, AFS results, and other variables did not show statistical significance in relation to MDR-TB occurrence. It is important to note that the lack of statistical correlation in this study does not imply that these variables are unrelated to MDR-TB occurrence, as differences in study populations, sample sizes, and variables analyzed may account for these discrepancies. Nonetheless, we opted to focus on the variables that demonstrated statistical significance in predicting MDR-TB occurrence in this study.

Rural residents have a higher likelihood of developing MDR-TB compared to urban residents, a finding consistent with Desissa et al.’s research (18). Cai et al. (19) also support this conclusion through a systematic analysis of MDR-TB risk factors among Chinese residents. Several factors contribute to the increased risk of MDR-TB among rural residents. Firstly, rural areas have limited medical resources for TB prevention, screening, and treatment, potentially leading to delays in standardized TB treatment and the subsequent emergence and transmission of MDR-TB. Secondly, inadequate medical supervision in rural settings may result in individuals with TB not completing their treatment as prescribed by healthcare providers or resorting to non-standard anti-TB medications, thereby elevating the risk of MDR-TB. Thirdly, rural residents may possess a lower level of TB awareness and knowledge, leading to delays in seeking medical attention or non-adherence to treatment recommendations, consequently heightening the chances of MTB developing resistance to drugs like rifampicin and isoniazid. Lastly, the challenging living conditions in rural areas, characterized by poor living standards, may further contribute to the increased spread and incidence of MDR-TB.

It is widely acknowledged that individuals with TB who have undergone retreatment for TB are at a higher risk of developing MDR-TB compared to those who were initially treated. This conclusion has been supported by studies conducted globally, including in Central Africa, Malaysia, Europe, and Iran (20–23). The rationale behind this observation is that previous inadequate or irregular anti-TB treatment in retreated individuals with TB may not completely suppress MTB, allowing some MTB strains to adapt to the drug environment and develop drug resistance. Moreover, individuals with TB who discontinue medication upon symptom disappearance or due to poor compliance are at risk of treatment failure or recurrence, leading to persistent positive sputum cultures and ongoing lesions, significantly elevating the likelihood of secondary MDR-TB. This discovery underscores the importance for healthcare professionals to administer appropriate and effective treatment, as well as enhance monitoring and follow-up of individuals with TB.

Pulmonary cavity is a common clinical manifestation in individuals with TB who have normal immune function. However, individuals with low immune function may not exhibit cavities. The presence of cavities often suggests a more severe form of the disease and is a potential risk factor for MDR-TB. Global meta-analyses conducted by Xi et al. (13) and Wei et al. (24) both emphasize the significant association between pulmonary cavity and MDR-TB. The presence of pulmonary cavities provides an environment for MTB to persist and replicate, potentially leading to adaptation and resistance to anti-TB drugs, thus elevating the risk of MDR-TB. The presence of pulmonary cavities hinders the complete penetration of anti-TB drugs into the affected area, diminishing the efficacy of the treatment. Consequently, even with standard anti-TB therapy, complete eradication of the MTB may not be achieved, increasing the likelihood of drug resistance. Moreover, pulmonary cavities can disrupt local immune responses, compromising the body’s ability to clear the MTB and making individuals with TB more susceptible to infection by other drug-resistant strains or progression to MDR-TB.

The study found that individuals with TB with elevated levels of UA are at a higher risk of developing MDR-TB. However, there is a lack of large-scale studies to definitively establish a causal relationship between high UA levels and MDR-TB. Sun et al. (25) demonstrated that individuals with DR-TB had significantly higher UA levels compared to individuals with drug-sensitive TB, indicating an increased risk of MDR-TB in individuals with high UA. It is hypothesized that the elevated UA levels could be a common side effect of anti-TB medication, leading to treatment interruptions and ultimately increasing the likelihood of MDR-TB development. Individuals with TB who have lower levels of CRP are more likely to develop MDR-TB, while those with higher levels have a reduced risk. This could be attributed to higher CRP levels indicating an inflammatory response triggered by TB, facilitating early detection and treatment to prevent progression to MDR-TB. Furthermore, CRP levels serve as a marker for the overall immune status of the individual. The decreased CRP levels in individuals with MDR-TB may suggest underlying immunosuppression, which is a known risk factor for MDR-TB development.

In this study, a nomogram model was developed and validated using demographic information, clinical characteristics, laboratory and imaging indicators of individuals with DR-TB to predict the risk of MDR-TB. A higher AUC value demonstrated effective discrimination by the model, the results of the Hosmer-Lemeshow test indicated satisfactory calibration, and a larger threshold under the DAC curve signified practical clinical utility of the model. The model’s indices are easily accessible in clinical settings, making it a convenient tool for predicting MDR-TB risk. By forecasting the risk of MDR-TB and facilitating timely screening of high-risk individuals, individualized treatment plans can be implemented to minimize ineffective treatments, thereby improving cure rates and curtailing the spread of MDR-TB. However, there are limitations to this study: (1) being retrospective, there may be residual confounding factors; (2) data were sourced from a single medical institution, limiting generalizability; (3) the study’s indicators were restricted to existing variables, potentially introducing selection bias. Future prospective studies involving multiple medical centers should expand research variables to further validate the MDR-TB prediction model in individuals with DR-TB.

In summary, this study developed a nomogram model to predict the risk of MDR-TB in individuals with DR-TB based on factors such as residence, types of TB treatment, presence of pulmonary cavity, levels of UA and CRP. The model demonstrated good consistency, accuracy, and clinical utility, offering valuable insights for prompt diagnosis, treatment, and effective prevention and control of MDR-TB in clinical settings.

## Data Availability

The raw data supporting the conclusions of this article will be made available by the authors, without undue reservation.
